# Gastrokine-1, an anti-amyloidogenic protein secreted by the stomach, regulates diet-induced obesity

**DOI:** 10.1038/s41598-021-88928-8

**Published:** 2021-05-04

**Authors:** Anne-Marie C. Overstreet, Bernadette E. Grayson, Antonia Boger, Danika Bakke, Erin M. Carmody, Cayla E. Bales, Shirley C. Paski, Stephen F. Murphy, Christopher R. Dethlefs, Kara J. Shannon, Katie R. Adlaka, Claire E. Wolford, Vincent J. Campiti, Christina V. Raghunandan, Randy J. Seeley, David L. Boone

**Affiliations:** 1 Department of Microbiology and Immunology, Indiana University School of Medicine-South Bend, RCH122, 1234 N. Notre Dame Ave., South Bend, IN 46617 USA; 2 Division of Endocrinology, Diabetes and Metabolism, Department of Internal Medicine, University of Cincinnati, Cincinnati, OH USA; 3 Cedars-Sinai Medical Center, Los Angeles, CA USA; 4 Department of Urology, Feinberg School of Medicine, Northwestern University, Chicago, IL USA; 5 Department of Biology, University of Notre Dame, South Bend, IN USA; 6 Present Address: Department of Neurobiology and Anatomical Sciences, University of Mississippi Medical Center, Jackson, MS USA; 7 Present Address: Department of Surgery, University of Michigan Health System, Ann Arbor, MI USA

**Keywords:** Stomach, Obesity, Microbiome

## Abstract

Obesity and its sequelae have a major impact on human health. The stomach contributes to obesity in ways that extend beyond its role in digestion, including through effects on the microbiome. Gastrokine-1 (GKN1) is an anti-amyloidogenic protein abundantly and specifically secreted into the stomach lumen. We examined whether GKN1 plays a role in the development of obesity and regulation of the gut microbiome. Gkn1^−/−^ mice were resistant to diet-induced obesity and hepatic steatosis (high fat diet (HFD) fat mass (g) = 10.4 ± 3.0 (WT) versus 2.9 ± 2.3 (Gkn1^−/−^) *p* < 0.005; HFD liver mass (g) = 1.3 ± 0.11 (WT) versus 1.1 ± 0.07 (Gkn1^−/−^) *p* < 0.05). Gkn1^−/−^ mice also exhibited increased expression of the lipid-regulating hormone ANGPTL4 in the small bowel. The microbiome of Gkn1^−/−^ mice exhibited reduced populations of microbes implicated in obesity, namely Firmicutes of the class Erysipelotrichia. Altered metabolism consistent with use of fat as an energy source was evident in Gkn1^−/−^ mice during the sleep period. GKN1 may contribute to the effects of the stomach on the microbiome and obesity. Inhibition of GKN1 may be a means to prevent obesity.

## Introduction

The number of obese and overweight Americans over the age of 20 exceeded 70% in 2013–2014 with obesity rates exceeding 37% at that time^1^. Obesity is a risk factor for a range of diseases including nonalcoholic steatohepatitis, cancer, diabetes, cardiovascular disease, hypertension, arthritis and others^2^. The cure for obesity is diet and exercise and in some cases bariatric surgery. These interventions can lead to weight loss but many patients find it very difficult to successfully lose weight or to maintain a successful weight reduction. Thus, therapies to promote or maintain successful weight loss are needed. One barrier to the development of weight-loss therapies is our incomplete understanding of the physiological regulators of diet-induced obesity. A better understanding of factors that regulate metabolism and accumulation of body fat may help in the development of drugs to fight obesity.

The stomach, which is a hollow muscular organ that initiates the second stage of digestion, regulates body weight and metabolism^3^. The stomach can directly impact appetite through the production of the orexigenic hormone ghrelin^4^. The effectiveness of bariatric surgery in weight loss and metabolic syndrome, which can occur independent of mechanical restriction of food intake or malabsorption of calories, suggest that the stomach plays important roles in obesity and metabolism beyond its role in digestion^5^. The benefits of bariatric surgery can occur independent of ghrelin, suggesting that other additional stomach functions may contribute to the regulation of metabolism and obesity^6^.

Bariatric surgery results in short- and long-term changes in the gut microbiome of patients and this altered microbiome promotes reduced fat accumulation when transplanted into mice^7^. How the stomach contributes to regulation of the gut microbiome is not known.

Gastrokine-1 (GKN1) is a small protease-resistant protein that is made exclusively and abundantly in the stomach^8,9^. GKN1 is not a circulating hormone but is instead secreted into the gut lumen from gastric foveolar epithelial cells^8,10^. GKN1 is protease-resistant and very stable, refolding at elevated temperatures and high concentrations of urea^11^, suggesting that this secreted protein may be resistant to digestion and have effects in the intestine beyond the stomach. GKN1 is highly conserved in mammals and has a single domain called a BRICHOS domain^12–14^. Like other BRICHOS domain containing proteins, GKN1 is anti-amyloidogenic and can prevent formation of model amyloid fibers *in vitro*^15^. The physiological function of GKN1 is not known. Since it is abundantly and exclusively made in the stomach and secreted into the lumen where it can potentially interact with microbes, we hypothesized that GKN1 may play a role mediating the stomach’s effects on the microbiome, metabolism or body weight gain. To test this we generated GKN1^−/−^ mice and assessed their body composition and sensitivity to diet-induced obesity.

## Methods

All methods were carried out in accordance with relevant guidelines and regulations.

### Animal studies

All animal protocols were approved by the University of Notre Dame (Boone) or the University of Cincinnati (Seeley) Institutional Animal Care and Use Committees. All experiments were carried out in accordance with the ARRIVE (Animal Research: Reporting of In Vivo experiments) guidelines and in accordance with the NIH Guidelines for the Care and Use of Laboratory Animals. GKN1^+/−^ mice on a C57Bl/6 genetic background were generated by the trans-NIH Knockout-Out Mouse Project (KOMP) and bred and maintained in our facility on that background. Mice were provided food and water ad libitum and kept on a 12/12-h light/dark cycle. All cages contained at least one enrichment device in the form of a cardboard tube or “house”. To test the effects of a thermoneutral environment mice were maintained in a room kept at 30 °C. For normal chow diet, mice were maintained on Teklad diet 2919 global 19% protein-extruded rodent diet (22% calories from fat). For high-fat diet, mice were fed research diet D12492 (60% calories from fat).

### GKN1 measurement

RNA was extracted from tissues using TRIZOL (Thermo Fisher) and cDNA was generated using the High-Capacity cDNA Reverse Transcription kit (Applied Biosystems). GKN1 was amplified by 30 cycles of PCR using the primers listed in supplementary Table 1, and resolved by agarose gel electrophoresis.

Proteins were extracted from tissues using RIPA buffer and 30 μg total protein was resolved by SDS-PAGE using NuPAGE 4–12% precast gels and transferred to PVDF membranes (Thermo Fisher). Membranes were blocked with 5% non-fat milk, immunoblotted with rabbit anti-GKN1 antibody (1:2000 in 5% non-fat milk) (Proteintech) overnight at 4 °C, and developed with anti-rabbit IgG-HRP antibody. Actin immunoblotting and Ponceau red staining of membranes were used as controls. To assess Gkn1 protein in the gut lumen, contents were flushed in PBS, mechanically dissociated and filtered through nylon mesh and filtrates were precipitated in cold acetone, resuspended in Laemmli buffer and assessed by Western blotting.

Formalin fixed paraffin embedded (FFPE) 5 μm sections were deparaffinized to water and blocked in 2% horse serum before overnight incubation with rabbit anti-GKN1 antibody (Proteintech) (1:250 in 2% horse serum). Sections were washed and developed with anti-rabbit antibody using the ImmPRESS-HRP polymer detection kit (Vector Labs). Standard protocols were followed for H&E staining of FFPE tissues.

### Body composition and PET scans

Magnetic resonance imaging was used to assess whole body composition of live subjects using the EchoMRI system (EchoMRI, Houston, TX). To assess the formation of tumors, mice were injected i.v. with 200 μCi of (^18^F) fluorodeoxyglucose (Spectron MRC, South Bend, IN, USA) and scanned 30 min later with a trimodal ALBIRA PET/SPECT/CT image station (Carestream Health, Woodbridge, CT, USA) and analyzed as described^16^.

### Assessment of liver steatosis

Liver triglycerides were measured in approximately 50 mg of fresh frozen tissue using Pointe Scientific Triglyceride reagent set (#T7532-120) according to the manufacturer’s specifications. Histological assessment of fat in liver was performed on FFPE tissues using H&E staining. In addition, to visualize fat accumulation, OCT-embedded frozen sections were stained with oil red O. Sections of 8 mm were dried, fixed in 4% paraformaldehyde, pretreated with 60% isopropanol, stained in 0.3% oil red O (w/v in 60% isopropanol), de-stained in 60% isopropanol, counterstained in hematoxylin and photographed in aqueous media.

Liver RNA was collected in TRIZOL and qPCR was performed to assess the expression of gluconeogenesis genes (phosphoenolpyruvate carboxykinase (PepcK), glucose-6-phosphatase (G6P), fructose-1,6-bisphophatase (FBP), pyruvate carboxylase (PC)) and to assess inflammation (interleukin-1 beta (IL1β) and interleukin-6 (IL6)) using primers listed in supplementary Table 1.

### Analysis of stool fat and calorie absorption

For assessment of food caloric extraction, food intake was measured by weighing food daily and total stool was collected, dried and analyzed by bomb calorimetry (UAB small animal phenotyping core, Birmingham AL). Efficiency of calorie absorption was determined by the percent of calories consumed vs calorie content of stool.

Fecal lipid content was assessed as previously described^17^. Briefly, diet was laced with 2.5% sucrose polybehenate and animals allowed to feed for 2 days. Fecal samples were collected 24 h later. Fecal lipid content was assayed by gas chromatography of fatty acid methyl esters. Dietary lipid absorption was estimated using a ratio of total fecal fatty acids to sucrose polybehenate.

### Glucose tolerance and insulin tests

Blood glucose was measured with the Accucheck meter and strips (Roche, Indianapolis, IN) following an i.p. injection of 1.5 g/kg dextrose in un-anesthetized mice previously fasted for 4 h. Fasting plasma insulin was measured by ELISA (Crystal Chem Inc., Downers Grove, IL).

### GLP-1, CCK, GIP, leptin and ghrelin assays

For measurements of CCK, GIP, and ANGPLT4, tissue scrapings were collected from washed intestinal samples and processed for RNA isolation and assessment of gene expression by qPCR. Ghrelin mRNA levels were assessed from mucosal scrapings of the stomach. Leptin and GLP-1 levels in serum were assessed by ELISA (EMD-Millipore, Burlington, MA).

For immunolocalization of ghrelin and leptin in stomach tissue FFPE tissue sections were treated for antigen retrieval by boiling 10 min in sodium citrate buffer (10 mM sodium citrate, 0.05% Tween-20 pH6.0) and stained with monoclonal anti-Gkn1 (R&D Systems), and polyclonal anti-ghrelin or anti-leptin antibodies (4C overnight; Thermo-Fisher), washed and stained with FITC or PE-conjugated secondary antibodies, mounted anti-fade media (prolong gold with DAPI) and photographed using fluorescence microscopy.

### Metabolic phenotyping

Mice were individually housed in metabolic cages (TSE Systems, Chesterfield, MO) for indirect calorimetry. Mice were acclimated for 48 h prior to measurements and assessed for 48 h of oxygen consumption and carbon dioxide production and RER was calculated by the ratio of CO_2_ produced to O_2_ consumed. Energy expenditure was calculated as total EE (kJ h − 1)/kg body weight = [16.3 × VO2 (L h − 1) + 4.57 × VCO2 (L h − 1)]/body weight (kg).

### Tests for inflammation

Gross morphological examination of secondary lymphoid organs and histology of tissues was performed to identify signs of overt inflammation. Flow cytometry was used to analyze lymphocyte activation from spleens and the intestinal lamina propria, as described^18^.

### Microbiome analysis

Sequencing of the 16S ribosomal RNA gene V4-V5 region was performed at Argonne National Laboratories (Argonne, IL) using the Illumina MiSeq instrument (San Diego, CA). Sequences were analyzed with the Quantitative Insights into Microbial Ecology (QIIME) toolkit version 1.9.1^19^. Reads were quality filtered and demultiplexed using a PHRED score of 30. Quality filtering resulted in 544,839 high-quality sequences total. The quality filtered reads were clustered using the open reference OTU picking workflow against the April 2018 release of the SILVA database with a sequence identity of 97% and uclust used as the clustering algorithm. Chimeras were removed using ChimeraSlayer. Low confidence OTUs were removed. Samples with less that 5,000 reads were removed from the OTU table. Statistical analysis was performed using the PhyloSeq package in R.

### Statistics

Statistical analyses were performed with Prism software version 7 (Graphpad, LaJolla, CA). ANOVA was used for multiple comparisons with post-hoc Tukey’s test. Paired comparisons were performed using the Welch *t* test. A *P* value of < 0.05 was considered significant.

## Results

### GKN1^−/−^ mice have reduced body fat on a normal chow diet

GKN1 is a small (18 kDa) protein made exclusively and abundantly in the stomach^8^. We confirmed the stomach specific expression of GKN1 mRNA and protein by PCR, immunoblotting and immunolocalization (Supplemental Figure S1). GKN1 was expressed in the foveolar epithelial cells of the gastric mucosa where it is packaged in mucus granules and secreted^8^. Gkn1 protein could be found intact in the lumenal contents of the small bowel and colon (Figure S1). To understand the function of GKN1, GKN1^+/−^ mice were bred and maintained on a C57BL/6 N background. GKN1^−/−^ mice were born at Mendelian frequencies with no overt health conditions.

**Figure 1:**
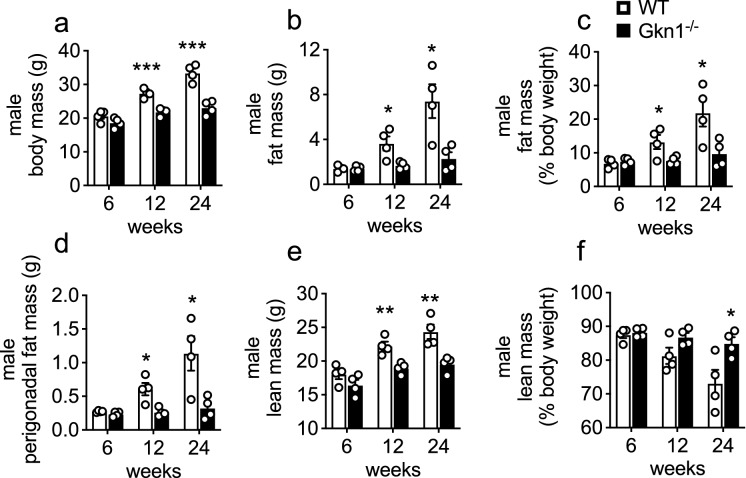
GKN1 regulates age-associated accumulation of body fat. Male WT and GKN1^−/−^ mice were maintained on NCD ad libitum and assessed at 6, 12 and 24 weeks of age for (**a**) body weight and (**b**) fat mass (by qMRI) or (**c**) fat mass as a percent of body weight. (**d**) Perigonadal fat pads were excised and weighed. (E&F) lean mass was also assessed by qMRI. *p < 0.05, **p < 0.01, ***p < 0.001 n = 4.

C57BL/6 mice are prone to diet-induced obesity^20,21^. When fed normal chow diet (NCD; Teklad 2019; 9% fat w/w; 22% calories from fat), male GKN1^−/−^ mice initially exhibited body weights comparable to WT littermates at 6 weeks of age (Fig. 1a). By 12 and 24 weeks of age, the GKN1^−/−^ mice weighed significantly less than their WT littermates (Fig. 1a). Body composition analysis using quantitative magnetic resonance (qMR) indicated that GKN1^−/−^ mice had significantly reduced levels of total body fat at 12 and 24 weeks of age (Fig. 1b,c) and this was confirmed by weighing individual fat depots (Fig. 1d). GKN1^−/−^ mice also displayed significantly reduced absolute lean mass (Fig. 1e). This was not lower when expressed as a percent of total body mass, as GKN1^−/−^ mice had a higher percentage of lean mass compared to WT littermates (Fig. 1f). This reduced body fat phenotype was evident in both male and female GKN1^−/−^ mice (Fig. 1 and Supplemental Figure S2). Thus, the absence of GKN1, a protein made and released into the stomach, results in reduced diet-induced accumulation of body fat.

### GKN1^−/−^ mice express increased intestinal ANGPTL4

**Figure 2:**
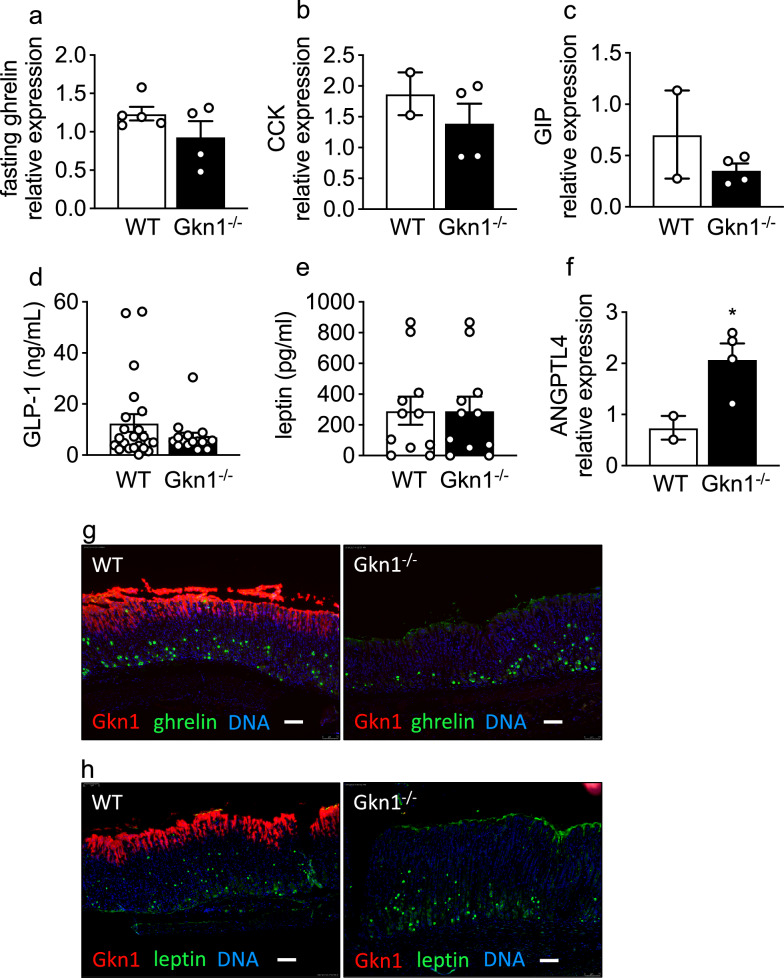
GKN1 regulates Angptl4 expression in the intestine. WT and GKN1^−/−^ mice were assessed for gastric mucosal expression of (**a**) ghrelin and intestinal gene expression of (**b**) CCK, (**c**) GIP mRNA measured by qPCR (n = 2–4). Serum levels of (**d**) GLP-1 or (**e**) leptin by ELISA (n > 4). (**f**) Intestinal expression of ANGPTL4 mRNA measured using qPCR (n = 2–4). The qPCR data was normalized to GAPDH. Immunolocalization of Gkn1 in the gastric mucosa compared to (**g**) ghrelin or (**h**) leptin in WT vs. Gkn1^−/−^ mice (scale bar is 75 μM). *p < 0.05.

To begin to understand how GKN1, a protein that is secreted into the gut lumen impacts peripheral fat levels, we assessed the expression of a variety of gut-expressed factors that regulate satiety, insulin and metabolism. The gastric expression of ghrelin and intestinal expression of CCK, GIP and circulating levels of GLP-1 and leptin were not significantly different in GKN1^−/−^ vs WT mice (Fig. 2a–e). Localization of ghrelin and leptin in the stomach was restricted to pit/neck cells (as opposed to Gkn1 localization in foveolar/mucus cells) and was not different between WT and Gkn1^−/−^ stomachs (Fig. 2g,h). The expression of angiopoietin-like 4 (ANGPTL4; aka FIAF) however, was significantly elevated in the intestinal tissue of GKN1^−/−^ mice compared to WT mice (Fig. 2f). This suggests that one possible role of GKN1 is to suppress intestinal expression of ANGPTL4, a microbially regulated factor produced in the intestine and known to regulate lipid metabolism in peripheral fat depots^22,23^.

### Altered small bowel microbiome of GKN1^−/−^ mice

**Figure 3:**
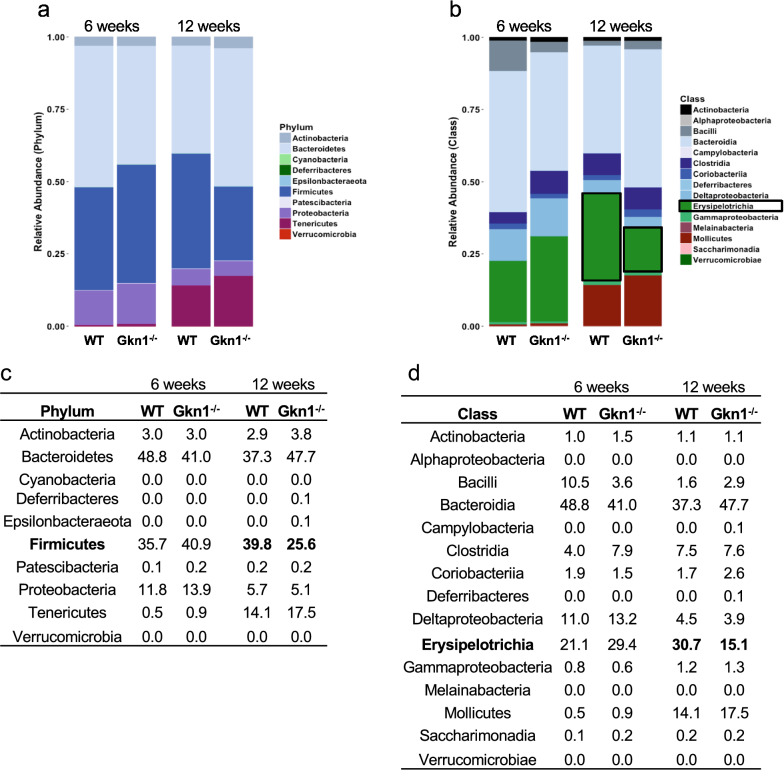
GKN1 regulates the small bowel microbiome. (A-D) Phylogenetic analysis of small bowel microbial populations in WT and GKN1^−/−^ mice at 6 and 12 weeks of age on NCD. (**a,c**) Relative phyla abundance in the small bowel of mice showing significantly reduced abundance of Firmicutes in 12-week, but not 6-week-old GKN1^−/−^ mice (bolded numbers are *p* < 0.01 n = 7). (**b**,**d**) Relative class abundance in the small bowel of mice showing decreased abundance of Erysipelotrichia in 12-week but not 6-week-old mice (bolded numbers are *p* < 0.01 n = 7).

The gut microbiome regulates obesity in mice and also potentially in humans^24–26^. ANGPTL4 expression in the gut is controlled by gut microbes such that germ-free mice exhibit high ANGPTL4 expression and are resistant to diet induced obesity^23^. Microbial colonization of germ-free mice suppresses ANGPTL4 expression^23^. Given that we observed altered ANGPTL4 expression in GKN1^−/−^ mice and that GKN1 is a luminal gut protein, we tested whether the gut microbiome is different between WT and GKN1^−/−^ mice. We compared the small bowel microbiome of WT and GKN1^−/−^ mice at 6 and 12 weeks of age on NCD. At 6 weeks of age, when GKN1^−/−^ and WT mice have comparable adiposity, there were no significant differences, at the level of phylum or class, in the populations of microbes in the small intestine (Fig. 3a–d). However, at 12 weeks of age, when GKN1^−/−^ mice have significantly less adiposity than WT mice, a significant difference in the prevalence of microbes of the phylum Firmicutes was observed (Fig. 3a,c). Specifically, GKN1^−/−^ mice exhibited significantly reduced prevalence of Firmicutes compared to WT mice. The differences in Firmicutes could be accounted for largely by differences in abundance of microbes in the class Erysipelotrichia, which were more abundant in the small intestinal microbiome of WT vs. GKN1^−/−^ mice at 12 weeks of age (Fig. 3b,d). Thus GKN1^−/−^ mice have an altered small bowel microbiome, which may contribute to resistance to diet induced obesity.

### GKN1^−/−^ mice are resistant to high-fat diet-induced obesity

**Figure 4:**
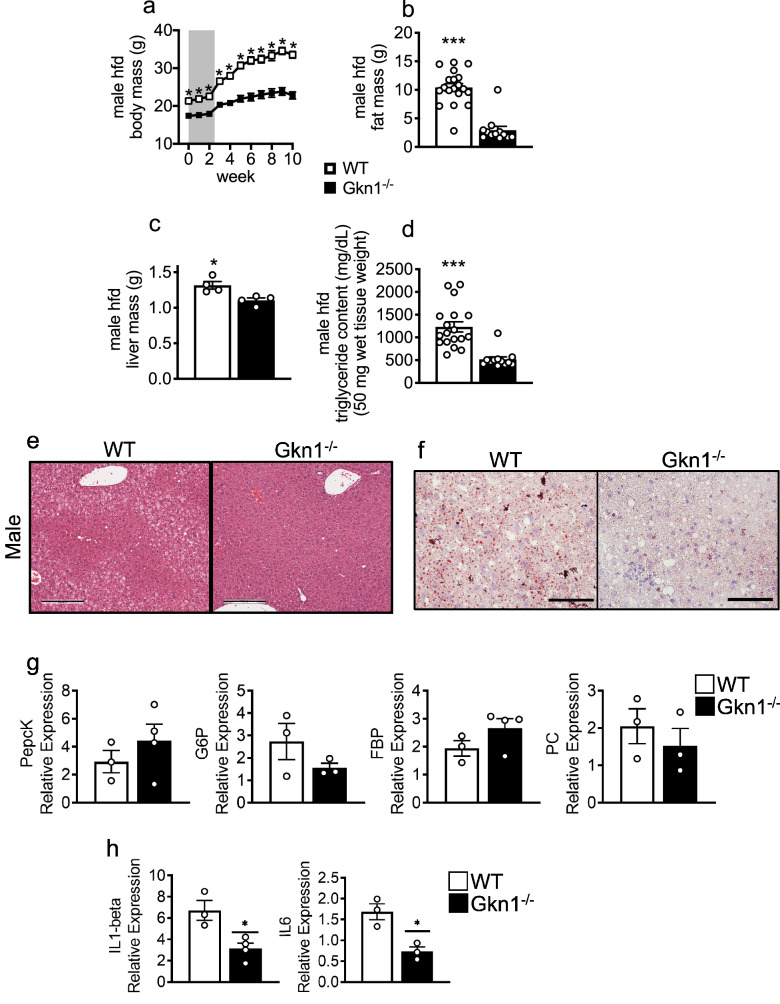
GKN1 regulates fat accumulation in response to high fat diet. Male WT and GKN1^−/−^ mice were individually caged and then switched from NCD to HFD and monitored for 8 weeks for (**a**) body weight and (**b**) fat mass (by qMRI) (n = 12–15). Livers were excised and assessed for (**c**) total weight, (**d**) triglyceride content and (**e**) presence of steatosis by H&E histology or presence of lipid by (**f**) staining with oil red O (scale bar is 200 μM). Expression levels of gene for (**g**) gluconeogenesis (PepcK, G6P, FBP, PC) or (**h**) inflammation (IL1b, IL6) were assessed in liver tissue from HFD treated mice. (n = 3–4) *p < 0.05, **p < 0.01, ***p < 0.001.

To test whether GKN1 is required for high fat diet (HFD)-induced obesity, mice were switched from NCD to HFD (Research Diets D12492; 35% fat w/w; 60% calories from fat) for 8 weeks. WT mice gained significant amounts of both weight and fat mass on this diet compared to GKN1^−/−^ littermates (Fig. 4a,b). As is typical of mice on HFD, WT mice developed signs of hepatic fat accumulation, which was significantly attenuated in GKN1^−/−^ mice (Fig. 4c–e). Liver mass and levels of hepatic triglycerides were significantly reduced in HFD fed GKN1^−/−^ mice compared to WT littermates (Fig. 4c,d). This reduced liver adiposity in HFD diet fed GKN1^−/−^ mice was also evident histologically (Fig. 4e,f). Although no changes in hepatic gluconeogenesis gene expression were observed between WT and Gkn1^−/−^ mice, there were significantly decreased levels of IL1β and IL6 mRNA in Gkn1^−/−^ suggesting that the resistance to steatosis was accompanied by reduced hepatic inflammation in Gkn1^−/−^ mice (Fig. 4g,h). livers Similar findings were observed in female GKN1^−/−^ mice (Supplemental Figure S3). Thus GKN1^−/−^ mice are resistant to HFD-diet induced obesity and hepatic steatosis.

### GKN1^−/−^ mice are healthy with no evidence of gastric cancer, inflammation or diabetes

**Figure 5:**
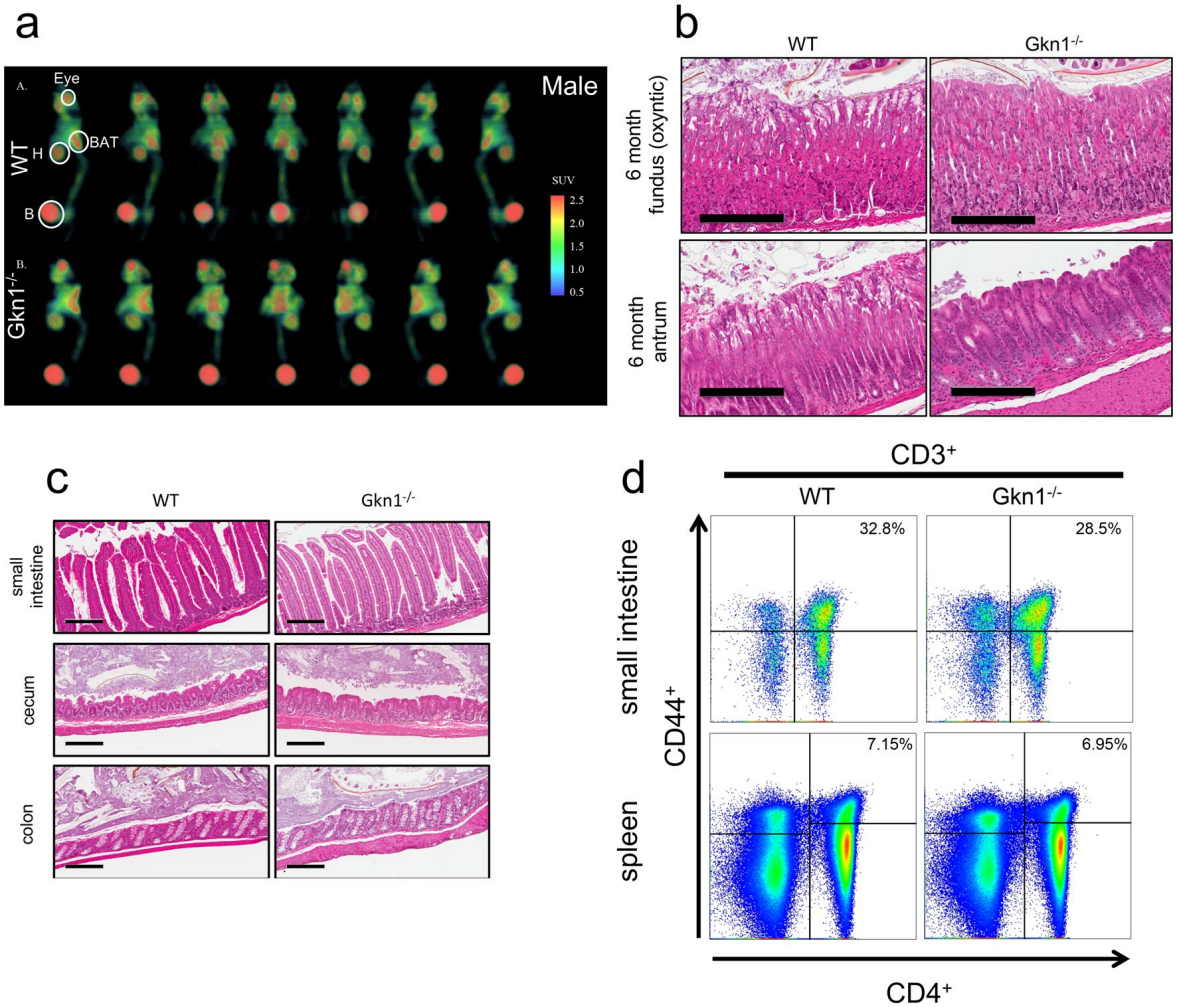
GKN1^−/−^ do not exhibit signs of cancer or inflammation. (**a**) Male WT and GKN1^−/−^ were imaged using PET scans. Highlighted areas are as follows: BAT – brown adipose tissue, H – heart, B – bladder. (**b**) Histology of aged (6 month old) antrum and fundus in WT and GKN1^−/−^ mice. (**c**) Histology of the small intestine, cecum and colon of WT and GKN1^−/−^ mice. (**d**) Flow cytometry analysis of small intestine and spleen cells gates on CD44^+^ and CD4^+^ (n = 4–7).

There are a variety of potential pathogenic causes of a lean phenotype in mice, including cancer, inflammation, malabsorption, diabetes, reduced food consumption and so on. We therefore assessed GKN1^−/−^ mice for alterations in a variety of potential lean phenotype inducing features. Although loss of GKN1 has been associated with gastric cancer^9,27,28^, we observed no increased prevalence of gastric or other cancer in GKN1^−/−^ mice up to 12 months of age (data not shown). This was confirmed by PET scans of GKN1^−/−^ mice indicating no cancers present, and by histology of the stomach and other tissues (Fig. 5a–c). A lean phenotype can result from inflammation but we observed no evidence of inflammation in GKN1^−/−^ mice by histology of multiple tissues nor by flow cytometric analysis of lymphocytes from the intestine or spleen (Fig. 5c,d). Similar phenotypes were observed in female mice (Supplementary Figure S4). Thus GKN1^−/−^ mice are lean but otherwise healthy with no signs of cancer or inflammation.

**Figure 6:**
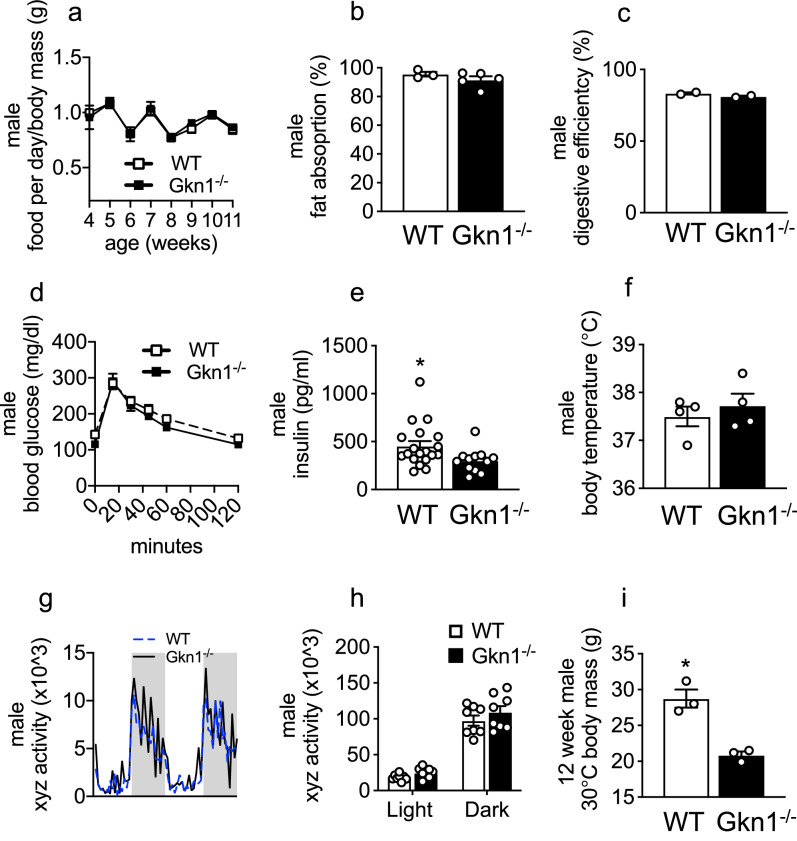
GKN1^−/−^ mice show no lean inducing phenotypes. WT and GKN1^−/−^ were monitored for (**a**) daily food intake (**b**) fat absorption (**c**) digestive efficiency (**d**) Blood glucose levels (**e**) insulin (**f**) body temperature (**G**,**h**) activity and (**i**) weight when raised under thermoneutral conditions (30 °C). *p < 0.05 (n = 2–19).

We also examined other potential causes of the lean phenotype of GKN1^−/−^ mice. We assessed whether GKN1^−/−^ mice consume less food than WT littermates and did not observe significant differences in food intake (Fig. 6a). We tested whether GKN1^−/−^ mice malabsorb fat or calories from their diet. We found that fat and calorie absorption were unchanged in GKN1^−/−^ mice compared to WT littermates (Fig. 6b,c). We next examined whether GKN1^−/−^ mice might be less fat due to diabetes. Glucose tolerance tests were normal in GKN1^−/−^ mice and fasting insulin levels were not elevated (in fact there was a significant decrease of fasting insulin in GKN1^−/−^ mice). These results indicate that GKN1^−/−^ mice are not diabetic (Fig. 6d,e). We also examined whether GKN1^−/−^ mice exhibit increased activity or body temperature that could explain the lack of fat accumulation. We found that body temperature and day/night locomotor activity were unchanged in GKN1^−/−^ mice (Fig. 6f–h). It is possible that the reduced adiposity of GKN1^−/−^ mice could be due to increased consumption of energy due to increased prevalence of brown or beige fat. We tested whether GKN1^−/−^ mice have elevated levels of thermogenic brown or beige fat. Peripheral fat depot gene expression of beige or brown fat genes was not increased GKN1^−/−^ mice compared to WT mice (Supplemental Figure S5). Lastly when housed at a thermoneutral temperature (30 °C) the reduced body weight of GKN1^−/−^ mice persisted, suggesting that altered thermogenesis or response to thermal stress do not account for the lean phenotype of GKN1^−/−^ mice (Fig. 6i). Similar findings were observed in female mice (Supplemental Figure S6). Thus, the lean phenotype of GKN1^−/−^ mice does not appear to be associated with reduced food intake, malabsorption of fat or calories, diabetes, elevated body temperature, increased locomotor activity or increased thermogenic fat.

### Increased resting fat metabolism in GKN1^−/−^ mice

**Figure 7:**
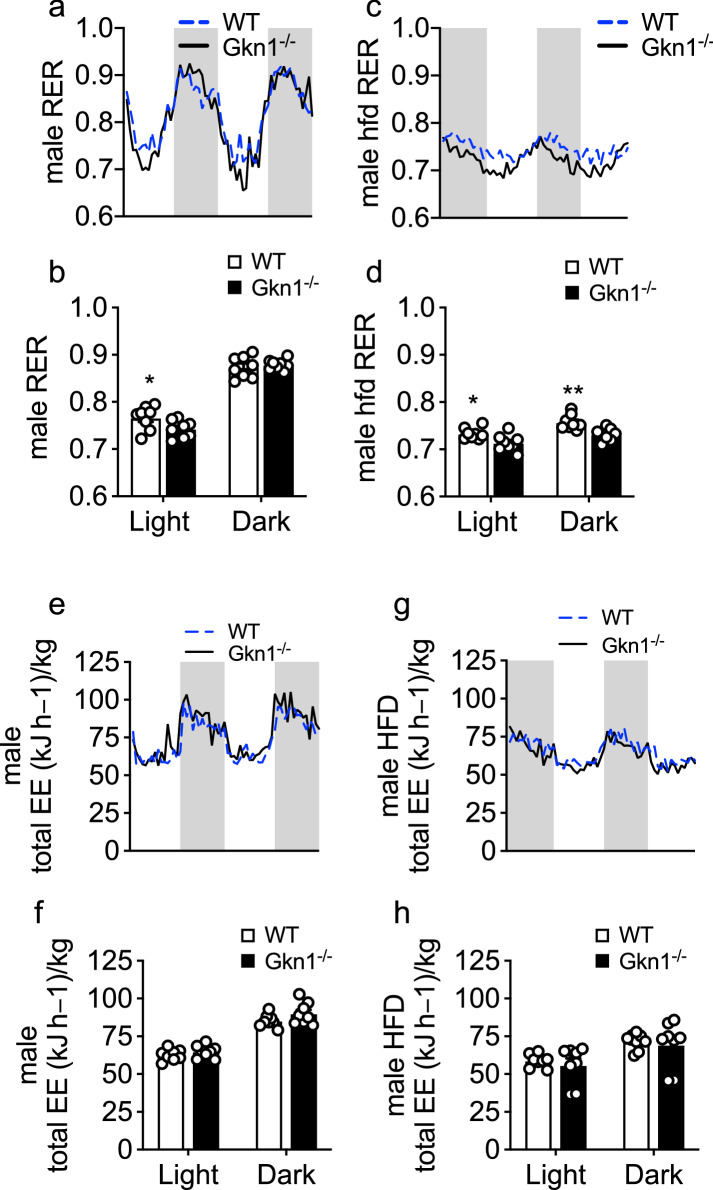
GKN1 regulates metabolism. Male WT and GKN1^−/−^ mice were individually housed in metabolic cages and monitored for CO_2_ production (VCO_2_) and O_2_ consumption (VO_2_) for 48 h with 12 hr cycles of light and dark and RER was calculated as VCO_2_/VO_2_. (**a**) Mice maintained on NCD show decreased RER during the light cycle (resting period) and increased RER during the dark cycle (active period) with (**b**) a lower average RER in GKN1^−/−^ mice (black line), compared to WT mice (blue line) during the light cycle. (**c**) Mice maintained on HFD showing a cyclical but blunted pattern of RER with (**d**) a lower average RER in GKN1^−/−^ mice (black line) compared to WT mice (blue line) during both light and dark cycles. The total body energy expenditure was not significantly different between WT and Gkn1^−/−^ mice on either the (**e**,**f**) NCD or the (**g**,**h**) HFD. In (**a**),(**C**),(**e**) and (**g**) the lines are the mean of 4 mice with error bars removed for clarity. *p < 0.05, **p < 0.01 n = 4.

To assess the metabolism of GKN1^−/−^ mice, we housed them in metabolic cages with continuous episodic monitoring of oxygen consumption and carbon dioxide production. This indirect calorimetry can be used as an index to distinguish the relative use of carbohydrates versus fat as the main energy source for metabolism. The respiratory exchange ratio (RER) is calculated using the amount of carbon dioxide produced compared to the amount of oxygen consumed. When carbohydrates are being used as the energy source, the RER approaches 1 whereas when fat is being used the RER is approximately 0.7. As expected, both WT and GKN1^−/−^ mice exhibited a diurnal pattern of RER such that during waking (feeding) hours the RER of mice on NCD was higher whereas during rest (less feeding) the RER decreased (Fig. 7). There was a significantly lower RER in GKN1^−/−^ mice on NCD during the resting period (light cycle) (Fig. 7a,b). This RER approached 0.7 suggesting a trend toward fat being used as the main energy source during the resting period of GKN1^−/−^ mice. On HFD, the diurnal variation of RER was blunted in both WT and GKN1^−/−^ mice but the trend continued such that GKN1^−/−^ mice exhibited a significantly lower RER during both the resting and awake periods (Fig. 7c,d). The total body energy expenditure was not significantly different between WT and Gkn1^−/−^ mice on a NCD (Fig. 7e,f), nor on a HFD (Fig. 7g,h) Similar findings were observed in female mice (Supplemental Figure S7). These data indicate that GKN1^−/−^ mice trend toward the use of fat as an energy source during the resting period and, when on a high fat diet, both during awake and resting periods.

## Discussion

The major conclusion and most notable finding of this study is that the absence of GKN1, a secreted, stomach-specific, anti-amyloidogenic protein, protects from diet-induced obesity. Specifically, GKN1 is required for the full development of excess body fat that occurs in mice with age on a normal chow diet, or subsequent to consuming a high fat diet. Since GKN1 is made exclusively in the stomach these findings identify an additional new mechanism for the regulation of diet-induced obesity by the stomach.

Previous studies have identified increased levels of microbes of the phylum Firmicutes, and particularly the class Erysipelotrichia, in high-fat diet-induced obesity and have shown that these microbes are causal for obesity^25,29–31^. In the present study we find that GKN1^−/−^ mice, which are resistant to obesity, have significantly lower levels of Firmicutes or Erysipelotrichia compared to WT mice, indicating that GKN1 plays a role in the development of an obesity-associated gut microbiome and suggests that GKN1 mediates obesity indirectly through effects on the gut microbiome. How GKN1 impacts the gut microbiome is not known, but as a secreted, lumenal protein one possibility is that GKN1 acts directly on intestinal microbes to alter their function. Microbes of all phyla produce functional extracellular amyloids that form fibers and are used to create biofilms that support a stable “niche” favoring the growth of microbes in the biofilm and protection from environmental challenges^32–34^. One potential mechanism of action of GKN1 in vivo could be the prevention of microbial biofilm formation, which could potentially broadly impact the microbiome. This might be comparable to the effects of dietary emulsifiers, which alter the gut microbiome to promote adiposity and metabolic syndrome^35^. By preventing biofilm formation, GKN1 may be acting as a natural “emulsifier” that regulates the microbiome to favor adiposity.

We observed increased expression of ANGPTL4 in the intestinal mucosa of GKN1^−/−^ mice. ANGPTL4 is a circulating factor that inhibits lipoprotein lipase and thus prevents uptake and storage of fat in peripheral adipose depots, allowing fat to be used as an energy source^22,23^. Thus, the reduced accumulation of fat in GKN1^−/−^ mice could potentially be due to increased intestinal expression of ANGPTL4 in these mice. Indirect calorimetry indicated that GKN1^−/−^ mice trend toward the use of fat as an energy source during sleep periods, which is consistent with the effects of ANGPTL4. Intestinal ANGPTL4 expression is regulated by the gut microbiome and therefore the effects of GKN1 on ANGPTL4 expression may be indirectly related to the effects of GKN1 on the microbiome^22,23^. In addition to decreased total and percent fat mass, Gkn1^−/−^ exhibited higher percent lean mass, compared to WT littermates. Since there is crosstalk between adipokines and myokines^36^ this raises the possibility that increased percent lean mass might also contribute to the reduced adiposity of Gkn1^−/−^ mice. Future studies of the crosstalk between adipokines and myokines in Gkn1^−/−^ mice might contribute to our understanding of obesity in this model.

The loss of GKN1 expression is associated with gastric cancer leading to the suggestion that GKN1 may be a tumor suppressor gene^9,27,28^. However, we found no evidence of gastric (or any other cancers) in GKN1^−/−^ mice. This suggests that loss of GKN1 alone is not sufficient to result in increased gastric cancer. It is possible that, since GKN1 is expressed in differentiated gastric epithelial cells, the loss of GKN1 in gastric cancer reflects the loss of epithelial cell differentiation. Indeed, at foci of gastric metaplasia in intestinal tumors and other sites, GKN1 is re-expressed^9^. Although we did not observe spontaneous gastric cancers in GKN1^−/−^ mice we cannot rule out the possibility that loss of GKN1 predisposes to gastric cancer in the context of other additional inducers such as inflammation or infection. In addition, the lifespan of mice extends to two years of age and so we cannot rule out the possibility that Gkn1^−/−^ mice might develop gastric cancer later in life.

GKN1 is highly conserved across evolution, suggesting that a similar role for GKN1 (and thus the stomach) in human obesity is possible^9,11^. From an evolutionary perspective, a protein that increases deposition of energy in fat depots would be advantageous. Further studies of the requirement for GKN1 in diets more comparable to human consumption will be informative. There are no described human patients with GKN1 deficiency or notable genetic associations of GKN1 with obesity. However, some conditions such as *Helicobacter pylori* infection or use of nonsteroidal anti-inflammatory drugs suppress GKN1 expression and it will be of interest to determine whether these impact metabolism or adiposity in ways consistent with those observed in GKN1^−/−^ mice^37,38^. Although controversial, *H. pylori* eradication is associated with increased BMI^39–41^ but whether this is related to restoration of GKN1 expression is not known. Other factors might explain increased BMI following *H. pylori* eradication, such as resolution of dyspepsia or normalization of gastric ghrelin expression resulting increased appetite. An association of NSAID use and BMI has not been reported, but high dose salicylates can result in lower serum insulin and glucose in diet induced obesity models, which phenocopies the insulin and glucose profile of GKN1^−/−^ mice^42^. Whether suppression of GKN1 by salicylates plays a role in the reversal of diet- and obesity-related insulin resistance remains to be determined. It is likely that bariatric surgery restricts access of GKN1 to the gut lumen and it will be of interest to determine if this plays some role in the beneficial effects of these procedures and in the changes in the gut microbiome that occur following bariatric surgery. Lastly, agents that suppress GKN1 expression or block its actions in the gut may have the potential to prevent diet-induced obesity, improving quality of life and reducing the burdens of obesity on health care.

## Supplementary information

Supplementary Information.
