# Supplementary material for: Gastrokine-1, an anti-amyloidogenic protein secreted by the stomach, regulates diet-induced obesity
Source: Sci Rep. 2021 May 4;11:9477. doi: 10.1038/s41598-021-88928-8 (PMC8096951; doi:10.1038/s41598-021-88928-8)

Supplementary table 1: Primers used for PCR and qPCR

Gkn1 FWD	5′- CGAGGAGATTCCAGGACCAA
Gkn1 REV	5′- AAGGACATCCGCAGAATCCA
CCK FWD	5′-GCACTGCTAGCGCGATACATC
CCK REV	5′-CCAGGCTCTGCAGGTTCTTAAG
GIP FWD	5′-GAGTTCCGATCCCATGCTAA
GIP REV	5′-TGTGCCTCTTTGTCCTCCTT
ANGPTL4 FWD	5′-CAATGCCAAATTGCTCCAATT
ANGPTL4 REV	5′-TGGCCGTGGGCTCAGT
Ghrelin FWD	5′-TCAAGCTGTCAGGAGCTCAGTA
Ghrelin REV	5′-TTGTCAGCTGGCGCCTCTT
PepcK FWD	5′-CCACAGCTGGTGCAGAACA
PepcK REV	5′-GAAGGGTCGATGGCAAA
G6P FWD	5′-CCATGCAAAGGACTAGGAACAA
G6P REV	5′-TACCAGGGCCGATGTCAAC
PC FWD	5′-GGACTCCTTTGGACACAGAG
PC REV	5′-AATCTCATTCTCATACACGTCG
FBP FWD	5′-CTGATATTCACCGCACTCTGG
FBP REV	5′-CGGCCTTCTCCATGACATAAG
IL1b FWD	5′-AACCTGCTGGTGTGTGACGTTC
IL1b REV	5′-CAGCACGAGGCTTTTTTGTTGT
IL6 FWD	5′-ACAACCACGGCCTTCCCTACTT
IL6 REV	5′-CACGATTTCCCAGAGAACATGTG
GAPDH FWD	5′- TGTCAAGCTCATTTCCTGGTATG
GAPDH REV	5′-TTGGGATAGGGCCTCTCTTG

Supplementary Figure 1

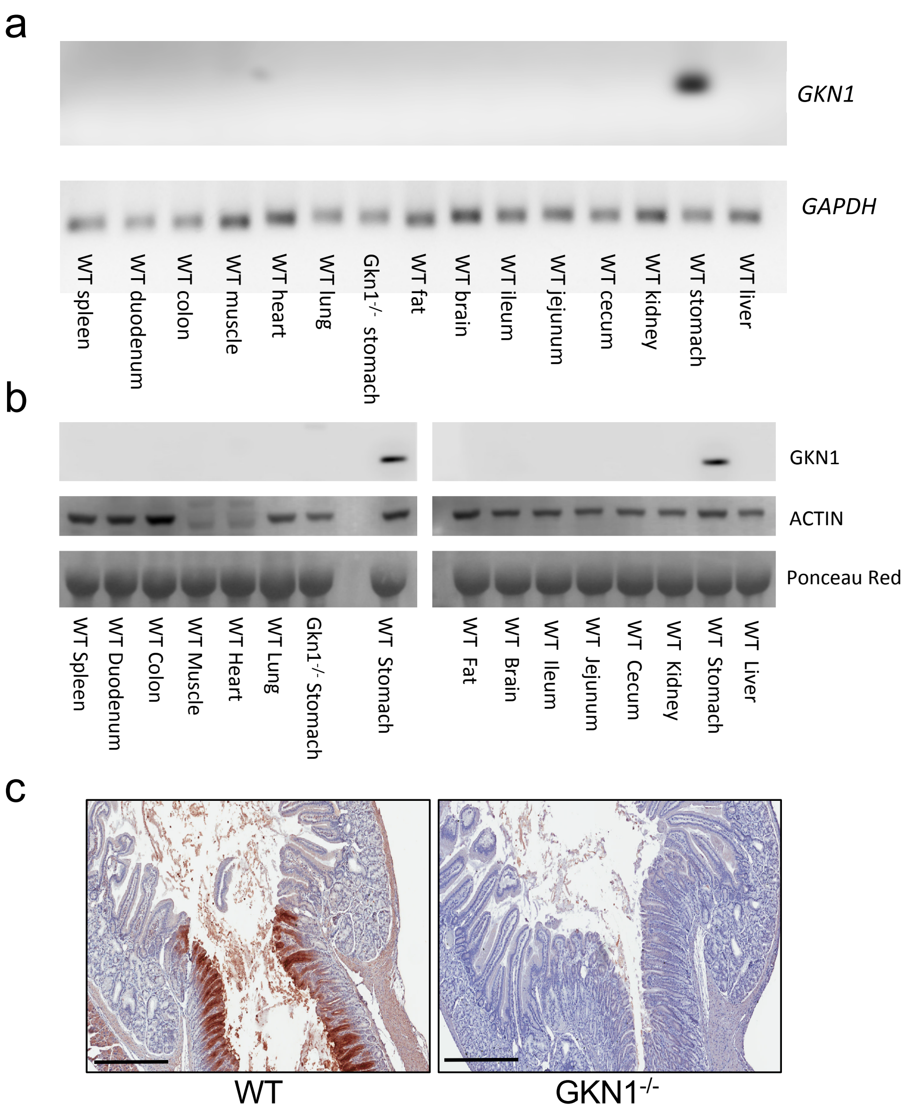

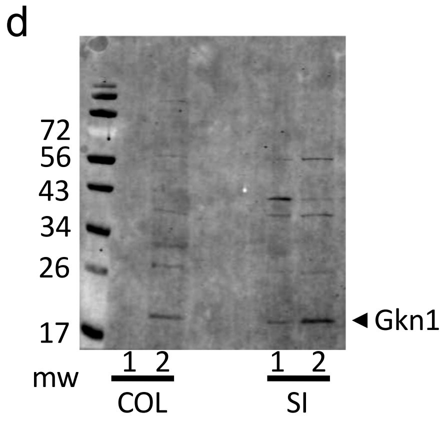

Supplemental Figure S1: Gkn1 RNA and protein expression is restricted to the stomach. (A) Inverted image of ethidium bromide stained gels showing reverse transcriptase PCR of Gkn1 mRNA from a variety of wild-type tissues showing Gkn1 expression only in the stomach (GAPDH as a control; gels are cropped to show only the regions with bands) . (B) Immunoblotting of proteins from a variety of tissues showing Gkn1 protein expression only in the stomach (Actin or Ponceau Red staining as controls; blots cropped to show only regions with bands). (C) Immunolocalization of Gkn1 protein in epithelial cells of the stomach showing demarcation where Gkn1 is not expressed in small bowel epithelium (control is the same staining of a Gkn1-/- stomach; scale bar = 500uM). (D) Immunoblot of gut lumenal contents showing Gkn1 protein (1: lysate of contents; 2: acetone precipitation of contents; blot cropped to remove lanes 8-10, which were not used). Uncropped immunoblots for S1B are shown in figure S8.

Supplementary Figure 2

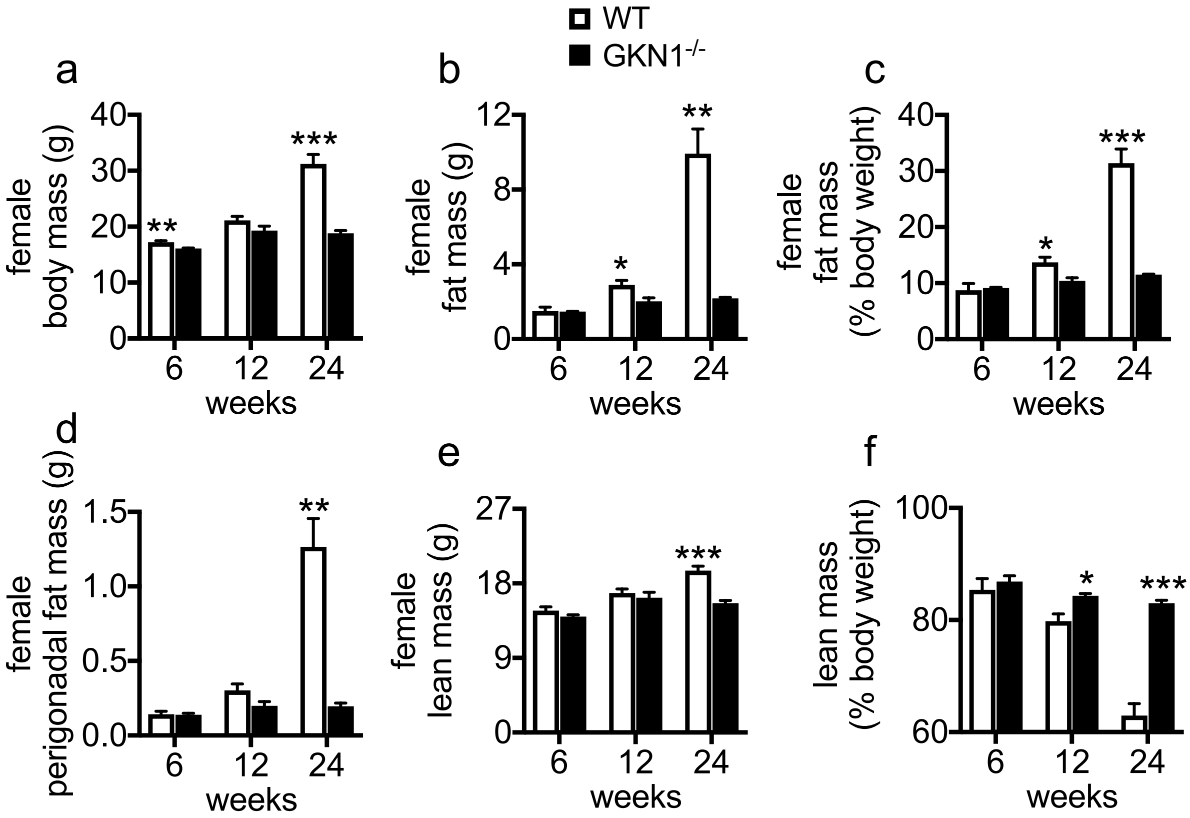

Supplemental Figure S2. GKN1 regulates age-associated accumulation of body fat in females. Female WT and GKN1$^{-/-}$ mice were maintained on NCD ad libitum and assessed at 6, 12 and 24 weeks of age for (A) body weight and (B) fat mass (by qMRI) or (C) fat mass as a percent of body weight. (D) Perigonadal fat pads were excised and weighed. (E&F) lean mass was also assessed by qMRI. *p<0.05, ** p<0.01, *** p<0.001 n=4.

Supplementary Figure 3

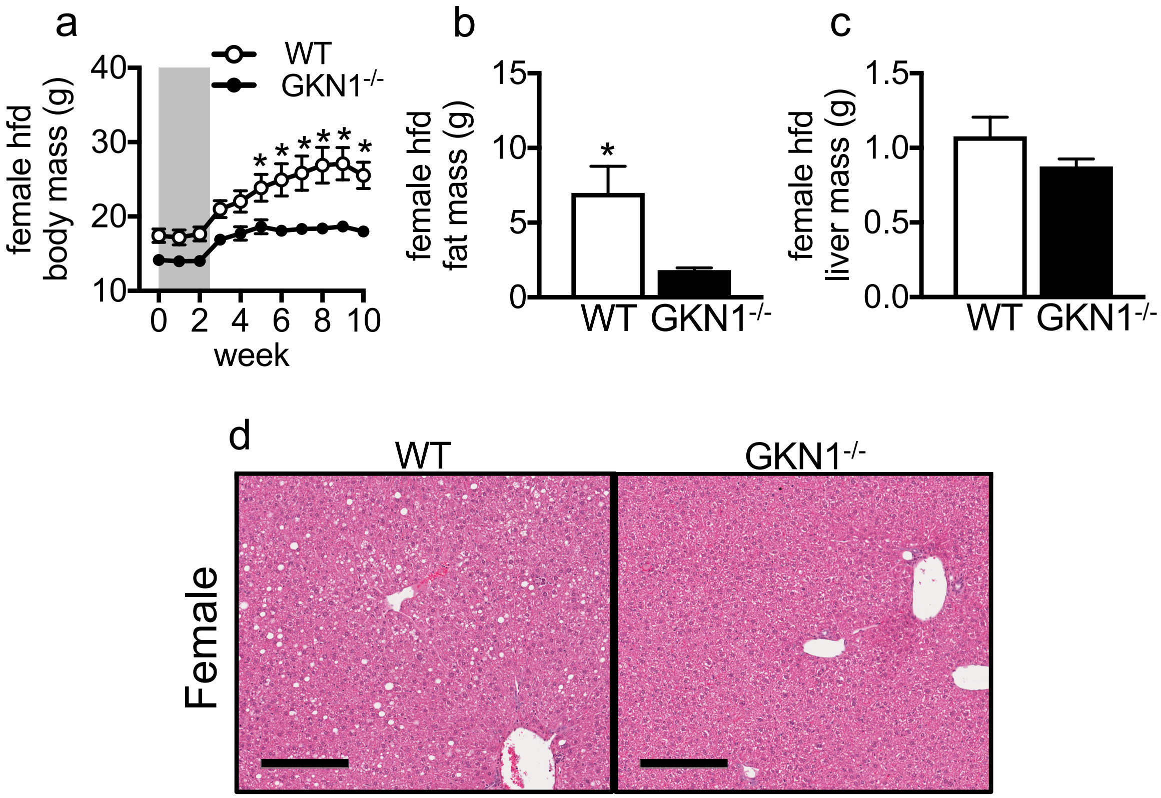

Supplemental Figure S3. GKN1 regulates fat accumulation in response to high fat diet in females. Female WT and GKN1$^{-/-}$ mice were individually caged and then switched from NCD to HFD and monitored for 8 weeks for (A) body weight and (B) fat mass (by qMRI) (n=12-15). Livers were excised and assessed for (C) total weight and (D) presence of steatosis by histology (n=4). *p<0.05

Supplementary Figure 4

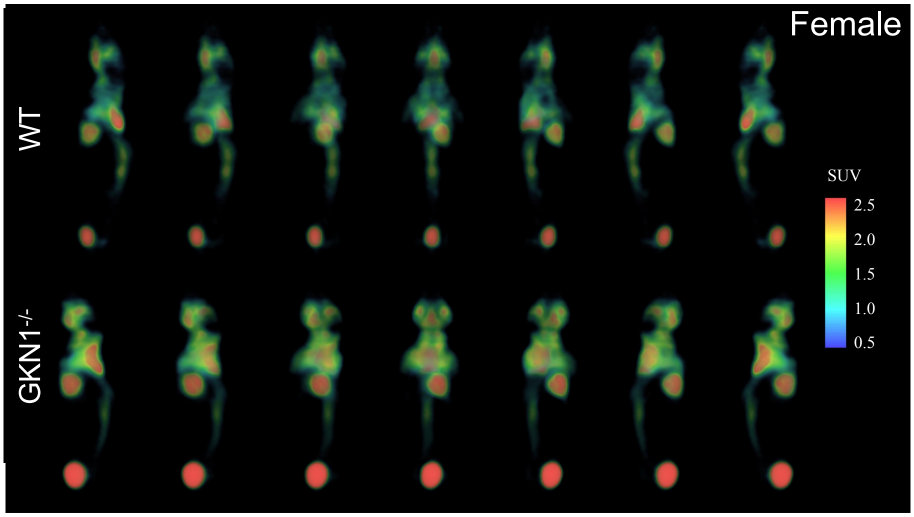

Supplemental Figure S4. Female GKN1$^{-/-}$ do not exhibit signs of cancer. Female WT and GKN1$^{-/-}$ were imaged using PET scans. Highlighted areas are as follows: BAT – brown adipose tissue, H – heart, B – bladder.

Supplementary Figure 5

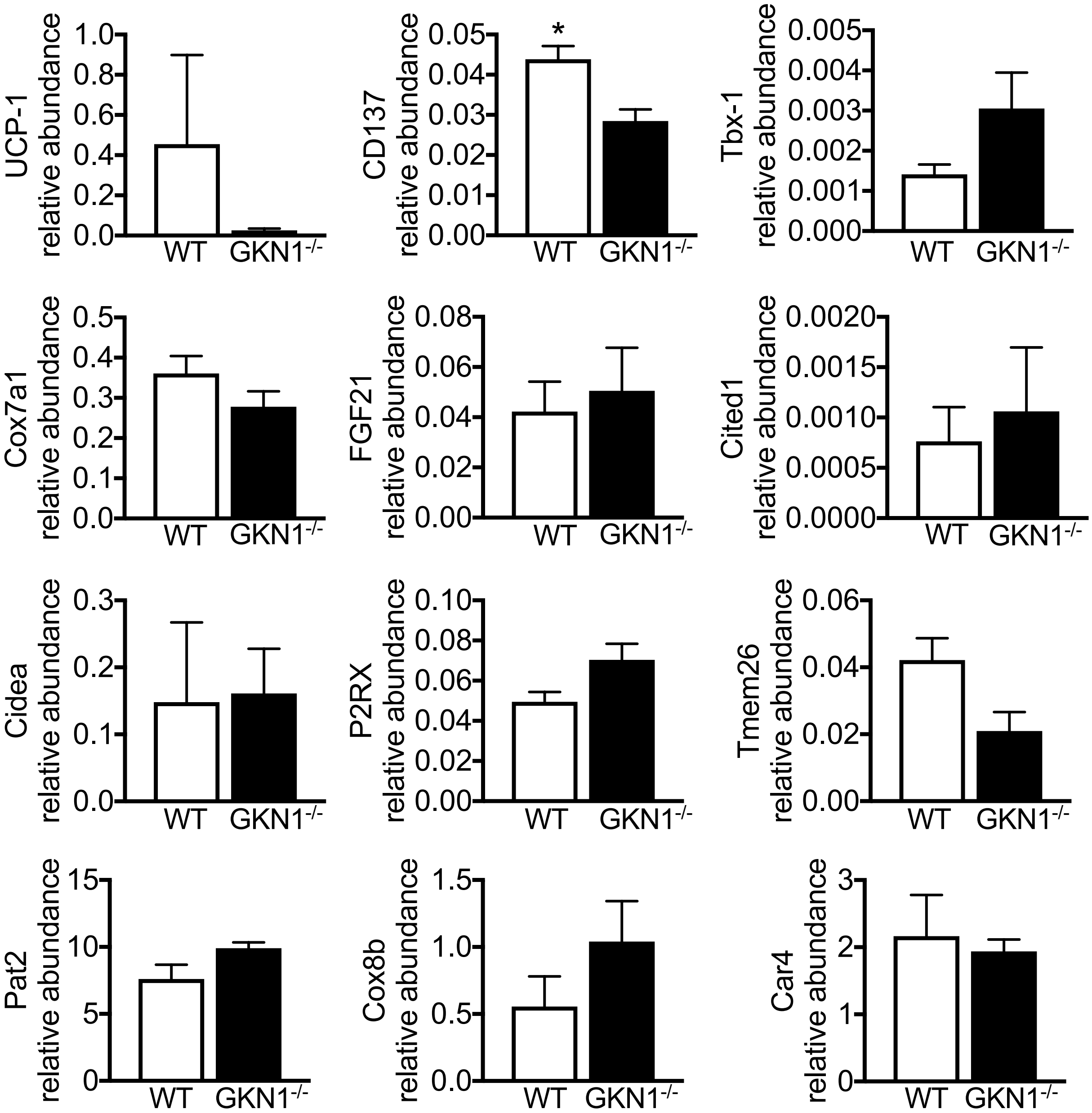

Supplemental Figure S5: Transcript analysis of fat does not indicate browning/beige-ing of white adipose tissue in Gkn1$^{-/-}$ mice. RNA extracted from inguinal fat pads was assessed for gene expression by qPCR. Transcripts for genes upregulated in brown/beige fat (UCP-1, CD137, Cox7a, FGF21, Cited1, Cidea, P2RX, TMEM26, Pat2, Cox8b and Car4) were not increased in adipose tissue of Gkn1$^{-/-}$ mice compared to WT mice. n>4, *p<0.05

Supplementary Figure 6

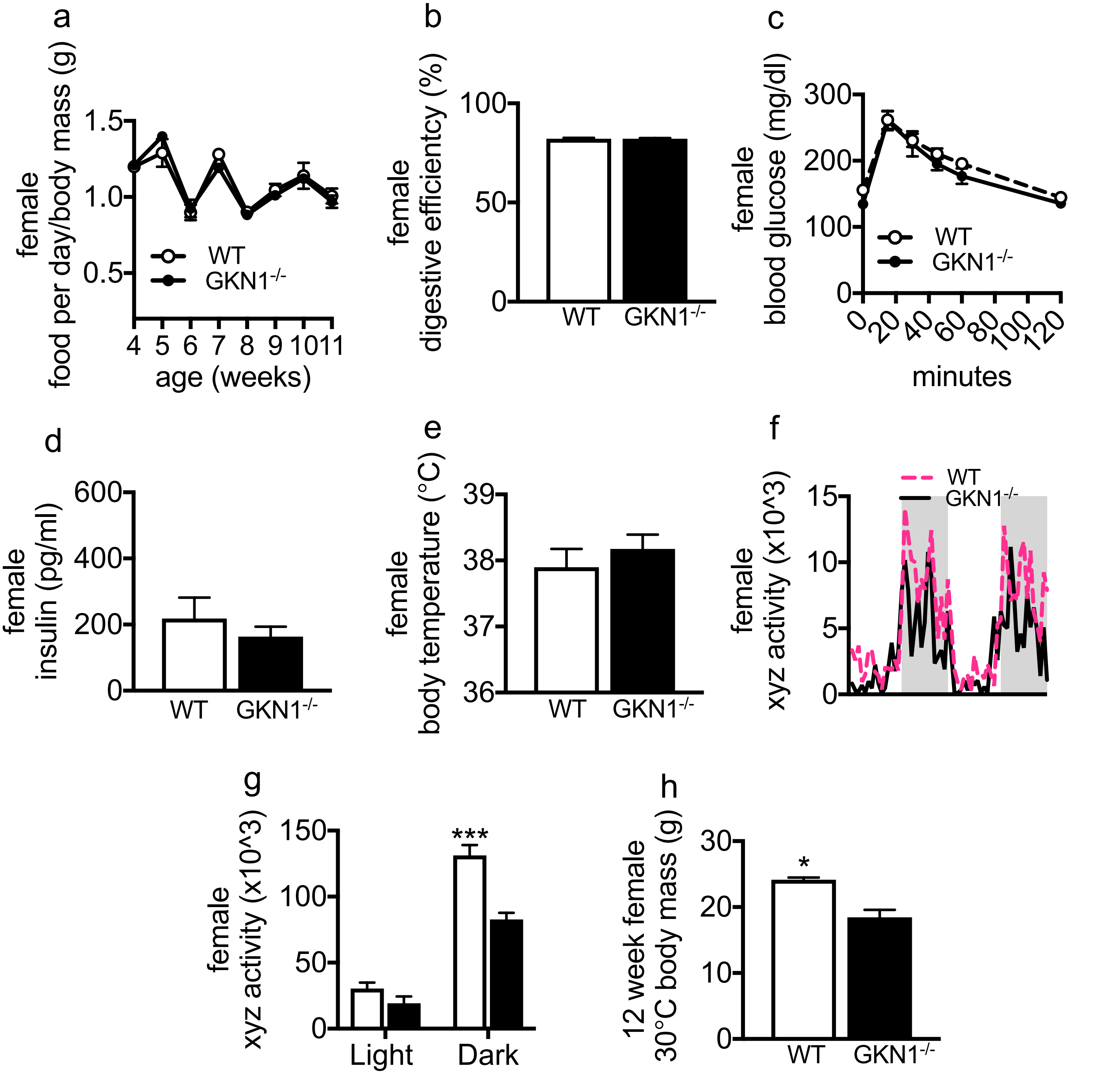

Supplemental Figure S6. Female GKN1$^{-/-}$ mice show no lean inducing phenotypes. WT and GKN1$^{-/-}$ were monitored for (A) daily food intake, (B) digestive efficiency (C) Blood glucose levels following oral challenge (D) fasting insulin (E) body temperature (F&G) activity and (H) body weight when raised under thermoneutral conditions (30°C). *p <0.05, ***p<0.005 (n=2-19).

Supplementary Figure 7

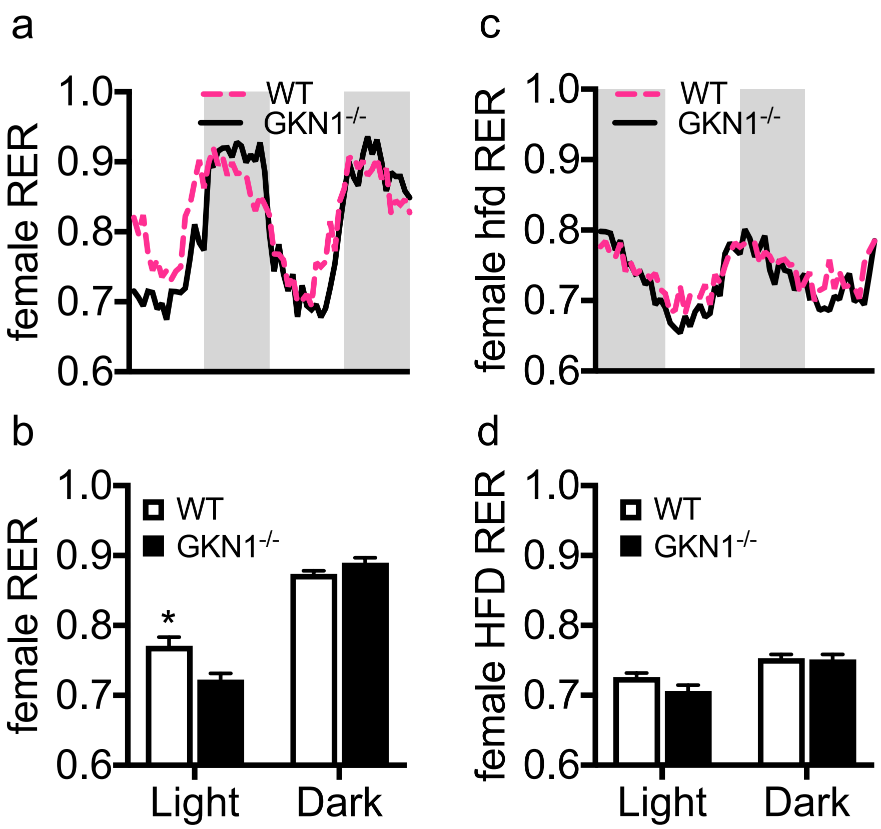

Supplemental Figure S7. GKN1 regulates metabolism in females. Female WT and GKN1$^{-/-}$ mice were individually housed in metabolic cages and monitored for CO_2 production (VCO_2) and O_2 consumption (VO_2) for 48 hours with 12hr cycles of light and dark and RER was calculated as VCO_2/VO_2. (A) Mice maintained on NCD show decreased RER during the light cycle (resting period) and increased RER during the dark cycle (active period) with (B) a lower average RER in GKN1$^{-/-}$ mice (black line), compared to WT mice (pink line) during the light cycle. (C) Mice maintained on HFD showing a cyclical but blunted pattern of RER with (D) a trend toward lower average RER in GKN1$^{-/-}$ mice (black line) compared to WT mice (pink line) during both light and dark cycles. In (A) and (C) the lines are the mean of 4 mice with error bars removed for clarity. *$p<0.05$ n=4.

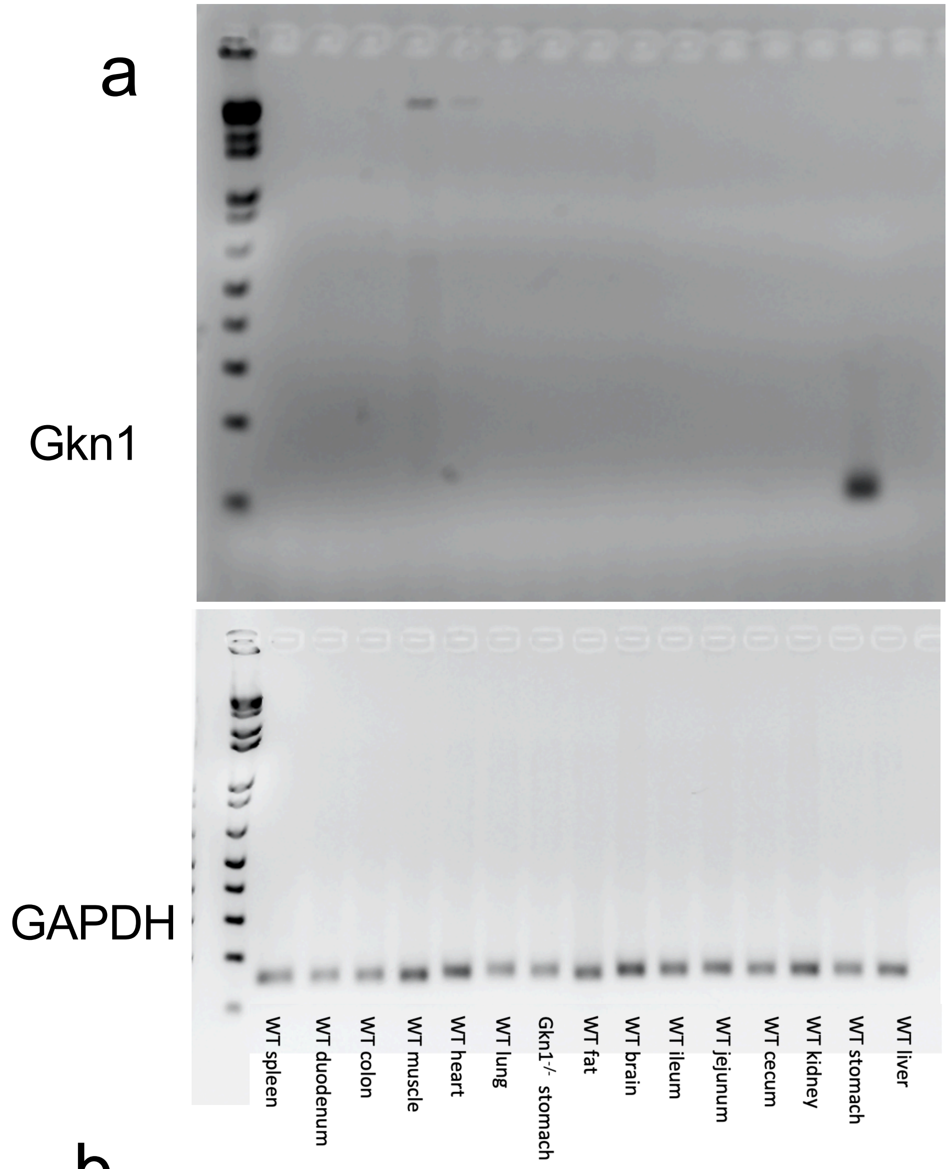

Supplemental Figure S8: Uncropped images of the blots used in Figure S1, showing (a) PCR products of Gkn1 and GAPDH from multiple tissues and (b) immunoblot for Gkn1 and actin, as well as total protein loaded (Ponceau red stain) from different mouse organs. (mw=Bio-Rad Precision Plus dual color molecular weight marker).

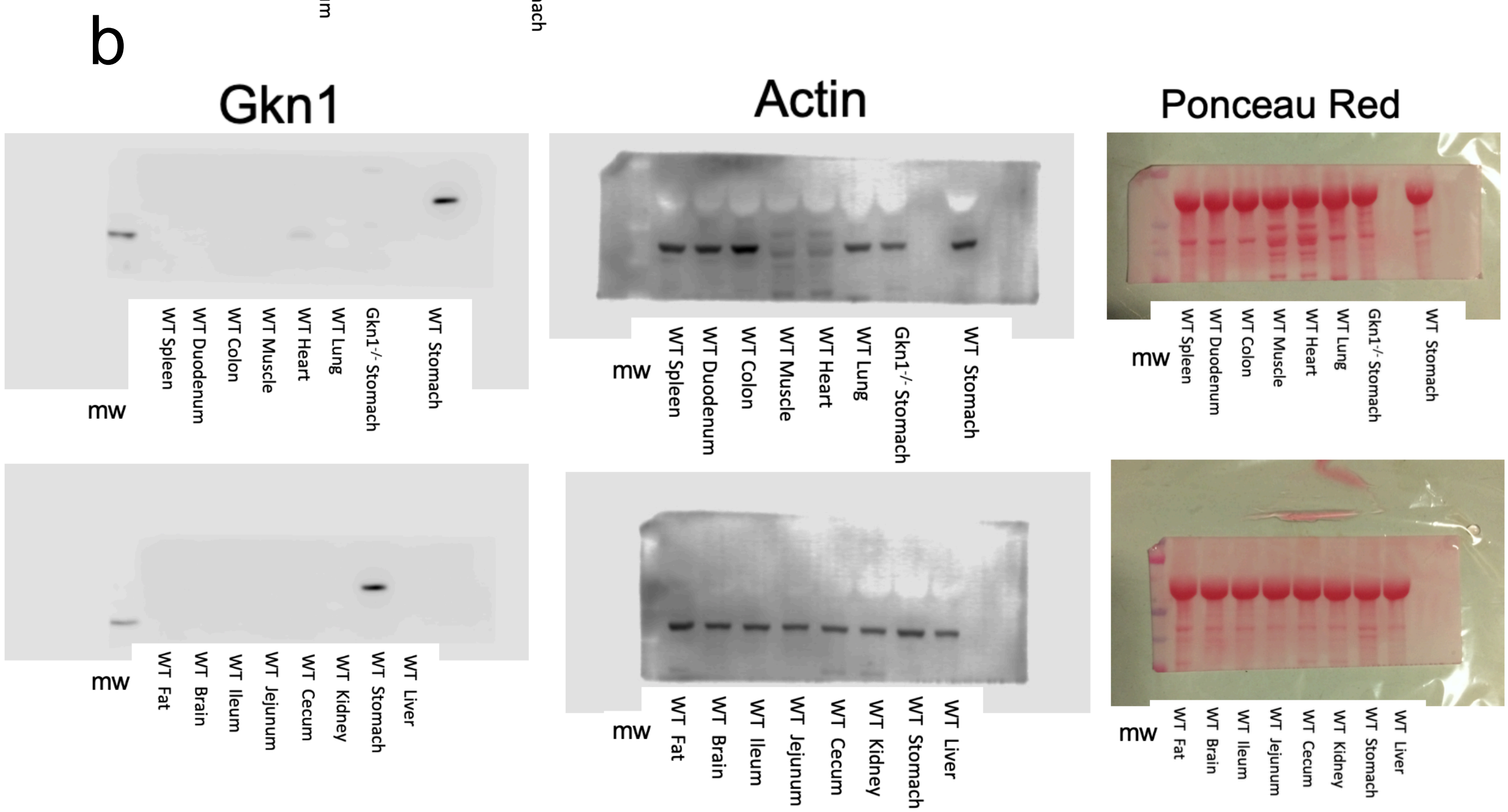

Supplement: Supplementary file 1 — Supplementary Information. [file 41598_2021_88928_MOESM1_ESM.pdf]
